# Giant Dissecting Aortic Aneurysm in an Asymptomatic Young Male

**DOI:** 10.1155/2015/958464

**Published:** 2015-02-23

**Authors:** Priyank Shah, Nishant Gupta, Irvin Goldfarb, Fayez Shamoon

**Affiliations:** ^1^St. Joseph's Regional Medical Center, 703 Main Street, Paterson, NJ 07503, USA; ^2^St. Michael's Medical Center, 111 Central Avenue, Newark, NJ 07102, USA

## Abstract

Giant aortic aneurysm is defined as aneurysm in the aorta greater than 10 cm in diameter. It is a rare finding since most patients will present with complications of dissection or rupture before the size of aneurysm reaches that magnitude. Etiological factors include atherosclerosis, Marfan's syndrome, giant cell arteritis, tuberculosis, syphilis, HIV-associated vasculitis, hereditary hemorrhagic telangiectasia, and medial agenesis. Once diagnosed, prompt surgical intervention is the treatment of choice. Although asymptomatic unruptured giant aortic aneurysm has been reported in the literature, there has not been any case of asymptomatic giant dissecting aortic aneurysm reported in the literature thus far. We report a case of giant dissecting ascending aortic aneurysm in an asymptomatic young male who was referred to our institution for abnormal findings on physical exam.

## 1. Introduction

Giant aortic aneurysm is a rare clinical entity. It is defined as aneurysm in the aorta exceeding 10 cm in its maximum diameter [1]. Since the risk of rupture is closely related to the size of the aneurysm, current guidelines recommend surgical intervention when the diameter of the aneurysm exceeds 5.5 cm [2]. The annual rate of rupture for aneurysms greater than 6 cm diameter is 14% [3]. Hence, a giant aortic aneurysm is not commonly seen in clinical practice. Atherosclerosis is one of the most common causes of aortic aneurysm. Other causes include Marfan's syndrome, giant cell arteritis, tuberculosis, syphilis, HIV-associated vasculitis, hereditary hemorrhagic telangiectasia, and medial agenesis [1]. We report a rare case of asymptomatic giant dissecting ascending aortic aneurysm in a young male.

## 2. Case Presentation

**Figure 1:**
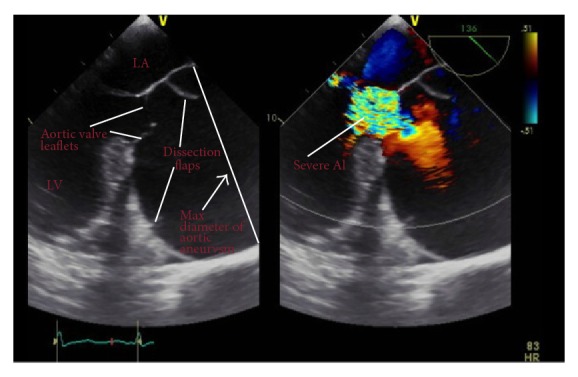
Transesophageal echocardiography in long axis aortic view showing giant aortic aneurysm (13 cm) and incidentally found dissection flaps in the ascending aorta (left). Also seen is severe aortic regurgitation (right). LA: left atrium, LV: left ventricle, and AI: aortic insufficiency.

**Figure 2:**
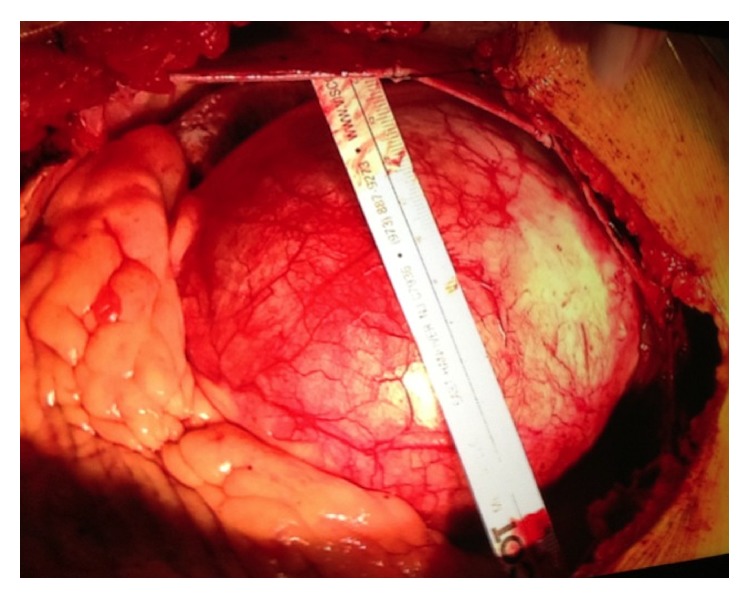
Gross image obtained during the Bentall procedure showing the giant ascending aortic aneurysm, 13 cm in diameter.

A 33-year-old asymptomatic Brazilian male with no significant prior medical history was referred to our institution for an outpatient transthoracic echocardiogram after his primary physician heard a diastolic murmur on a routine office visit. To our surprise, the transthoracic echocardiogram revealed a severely dilated proximal ascending aorta with effacement of aortic valve and aortic root. An immediate transesophageal echocardiogram was performed that revealed a proximal giant ascending aortic aneurysm of 13.0 cm in maximal diameter with well-defined dissection flaps on either side causing severe aortic insufficiency (Figure 1). However, aortic arch and descending aorta dimensions were within normal limits with preserved left ventricular systolic function. Findings were confirmed with CT angiography. Retrospectively when interviewed, patient endorsed a family history significant for Marfan's syndrome in his mother. On physical examination, the patient was noted to have some marfanoid features like buffalo hump and long arm span. On physical examination, there was a diastolic murmur, 3/4 in intensity in the aortic area. His heart rate was 82 beats/minute and blood pressure was 126/50 mm Hg. Rest of the examination was normal. The patient was then admitted to the hospital and underwent successful Bentall procedure constituting reconstruction of ascending aorta, aortic valve replacement, and reimplantation of coronaries.  Figure 2 shows gross specimen of the aneurysmal ascending aorta during surgery. Genetic testing in the patient revealed mutation in fibrillin-1 (FBN1) gene. The patient was counseled regarding genetic screening in first-degree family members. He was discharged from the hospital on day 7 after uneventful postoperative course on warfarin anticoagulation.

## 3. Discussion

The average rate of growth of thoracic aneurysms is 0.1–0.2 cm/year [1]. Giant aneurysms can have various presentations. The most feared complications include dissection and rupture. Leaking aneurysms may present with typical features of acute dissection with pericardial tamponade or with chest pain and collapse [1]. The risk of rupture depends on the size and the rate of growth of the aneurysm [4]. However, an asymptomatic giant thoracic aneurysm is extremely rare. There have been only two case reports in the literature of unruptured giant aortic aneurysm [4, 5]. Ours is the first case of an asymptomatic giant dissecting aortic aneurysm. By guidelines, our patient needed surgery even if there would not have been any dissection. Since our patient had dissection in the proximal portion of the aorta involving the root, valve sparing operation was not possible. Hence, he underwent Bentall procedure constituting reconstruction of ascending aorta, aortic valve replacement, and reimplantation of coronaries.

Young patients presenting with giant aortic aneurysm invariably have underlying genetic mutation, as was found in our patient. Aortic imaging is recommended for first-degree relatives of patients with thoracic aortic aneurysm and/or dissection to identify those with asymptomatic disease (class 1 indication, level of evidence B) [2]. If the mutant gene associated with aortic aneurysm and/or dissection is identified in a patient, first-degree relatives should undergo counseling and testing. Then, only the relatives with the genetic mutation should undergo aortic imaging (class 1 indication, level of evidence C) [2]. Hence, our patient underwent genetic testing and his first-degree relatives were also advised to undergo genetic testing since he tested positive for FBN1 gene mutation. This case also highlights the need for evaluation of diastolic murmurs as well as prompt evaluation of aortic root and ascending aorta at the time of diagnosis of Marfan's syndrome.
